# Green leafy vegetables from two Solanum spp. (*Solanum nigrum* L and *Solanum macrocarpon* L) ameliorate scopolamine‐induced cognitive and neurochemical impairments in rats

**DOI:** 10.1002/fsn3.628

**Published:** 2018-03-15

**Authors:** Opeyemi B. Ogunsuyi, Adedayo O. Ademiluyi, Ganiyu Oboh, Sunday I. Oyeleye, Abayomi F. Dada

**Affiliations:** ^1^ Department of Biomedical Technology Federal University of Technology Akure Nigeria; ^2^ Department of Biochemistry Federal University of Technology Akure Nigeria; ^3^ SLT Department (Biochemistry Unit) Federal Polytechnic Ede Ede Osun State Nigeria

**Keywords:** amnesia, functional food, neurochemistry, Solanum spp

## Abstract

This study examined the modulatory effect of Black nightshade (*Solanum nigrum* L) and African eggplant (*Solanum macrocarpon* L*)* leaves on cognitive function, antioxidant status, and activities of critical enzymes of monoaminergic and cholinergic systems of neurotransmission in scopolamine‐administered rats. Cognitive impairment was induced in albino rats pretreated with dietary inclusions of Black nightshade (BN) and African eggplant (AE) leaves by single administration (i.p.) of scopolamine (2 mg/kg body weight). Prior to termination of the experiment, the rats were subjected to spontaneous alternation (Y‐maze) test to assess their spatial working memory. Thereafter, activities of acetylcholinesterase (AChE), butyrylcholinesterase (BChE), monoamine oxidase (MAO), arginase, and antioxidant enzymes (catalase, SOD, and GST) of rat brain homogenate were determined. Also, the malondialdehyde (MDA), nitrite, and GSH contents of the homogenate were determined. The results showed that pretreatment with dietary inclusions of AE and BN significantly reversed the impairment in the rats’ spatial working memory induced by scopolamine. Similarly, elevations in activities of AChE, BChE, and MAO induced by scopolamine were significantly reversed in rats pretreated with dietary inclusions of AE and BN. In addition, impaired antioxidant status induced by scopolamine was reversed by pretreatment with dietary inclusions of AE and BN. This study has shown that dietary inclusions of AE and BN could protect against cognitive and neurochemical impairments induced by scopolamine, and hence, these vegetables could be used as a source of functional foods and nutraceuticals for the prevention and management of cognitive impairments associated diseases such as Alzheimer's disease.

## 1 INTRODUCTION

Several medicinal foods abound in traditional medicine with neuroprotective potentials that could be of importance in the management of several neurodegenerative diseases but with little or no scientific justification to substantiate their use. In the tropics, and especially in sub‐Saharan Africa, green leafy vegetables are used as one of the major components of local dishes. They are desired not only for being nutritive, but also for their folkloric reports of medicinal properties. African eggplant (AE) and Black nightshade (BN) are two Solanum species that serve mainly as vegetables for soup preparation in different parts of the world. Studies have reported potential health benefits of different parts of this vegetable. Juice extracted from the leaves of AE has been reportedly used in folklore for the treatment of the Parkinson's disease, memory impairment, angina, cancerous tumor, and inflammation among others (Haliński, Paszkiewicz, Gołębiowski, & Stepnowski, 2012; Oboh, Ekperigin, & Kazeem, 2005). BN leaves have also been reportedly used in traditional medicine for the management of several diseases including seizure and epilepsy, pain, ulcer, inflammation and diarrhea, some eye infections, and jaundice (Jain, Sharma, Gupta, Sarethy, & Gabrani, 2011), with reports of its use in tradio‐medical practices for neuromodulatory purposes but without scientific justifications. Due to the abundance of tropane alkaloids, the potential toxicity of these vegetables to humans is warned but only when consumed at large quantities (Haliński et al., 2012; Jain et al., 2011; Sánchez‐Mata, Yokoyama, Hong, & Prohens, 2010). Considering the increasing prevalence of neurodegenerative diseases such as Alzheimer's disease (AD) and Parkinson's disease (PD), studies into search for dietary sources of functional foods and nutraceuticals such as AE and BN, as well as their mechanisms of action, have become imperative.

Neurodegenerative diseases such as AD and PD are a group of chronic diseases, which are marked by systematic, selective, and continuous impairment of neuronal and cognitive functions (Martin, 1999). Each neurodegenerative disease is classified based on its specific clinical features of disease‐specific cellular biomarkers (Nieoullon, 2011). The AD is a neurodegenerative disease with features of cognitive impairments, decline in learning and memory functions, and impairments in attention, language, and spatial coordination (Klafki, Staufenbiel, Kornhuber, & Wiltfang, 2006). Pathophysiology of the AD includes impairments in cholinergic signaling, formation of toxic amyloid plaques, and neurofibrillary tangles in the brain, as well as oxidative neurotoxicity and inflammatory responses (Imbimbo, Lombard, & Pomara, 2005; Klafki et al., 2006). While the etiology of AD still remains an enigma, aging and genetic predispositions have been identified as major risk factors (Imbimbo et al., 2005). The epidemiological report has estimated the global prevalence of dementia to be up to 24 million, with a predictive doubling every 20 years until at least 2040 (Reitz, Brayne, & Mayeux, 2011). In addition, with over seven million new cases of dementia diagnosed annually across the world (Duthey, 2013), and as the global population ages, it is expected that the incidence of AD especially among the aged and in low‐ and middle‐income countries will increase (Duthey, 2013; Reitz et al., 2011). Several therapeutic drugs such as cholinesterase inhibitors have been designed for the management of AD, but their efficacy has been limited by attendant side effects (Oboh, Ogunsuyi, & Olonisola, 2017), coupled with the often rigorous daily routine of usage. Therefore, attention is being shifted to exploring natural plant products which can offer neuroprotective properties either acting singly (Oboh et al., 2017), or enhancing the therapeutic properties of conventional anti‐Alzheimer's drugs (Ogunsuyi, Adeoyo, & Oboh, 2017). In this regard, green leafy vegetables with medicinal properties have shown good potentials in both in vitro (Nwanna et al., 2016; Oboh et al., 2016) and in vivo studies (Baradaran, Rabiei, Rafieian, & Shirzad, 2012).

The use of the muscarinic cholinergic receptor antagonist scopolamine to induce animal models of amnesia and cognitive dysfunctions has been widely accepted (Akinyemi, Oboh, Oyeleye, & Ogunsuyi, 2017; El‐Khadragy, Al‐Olayan, & Abdel Moneim, 2014; Marisco et al., 2013). The ability of scopolamine to impair memory is characterized by dysfunction of the central cholinergic system of neurotransmission, impaired neuronal antioxidant status, and alteration in functional neurochemistry (Akinyemi et al., 2017; El‐Khadragy et al., 2014; Goverdhan, Sravanthi, & Mamatha, 2012). This has therefore made scopolamine a useful drug in simulating human dementia and AD (Akinyemi et al., 2017; Kwon, Kim, Lee, & Jang, 2009; Sadek, Khan, Darras, Pockes, & Decker, 2016).

In this study, we investigated the neuroprotective properties of African eggplant and Black nightshade by dietary inclusions (5% and 10%) of each vegetable sample in scopolamine‐induced cognitive impairment in rats. The spontaneous alternation (Y‐maze) test was used to evaluate the spatial working memory across various treatment groups, followed by an assessment of critical enzyme activities relevant to cognitive function in the brain tissue homogenates. We also investigated the brain nitrite level as a measure of nitric oxide content, as well as neuronal antioxidant status across various treatment groups. It is believed that findings from this study will be useful in dietary intervention and development of nutraceuticals for the management of cognitive dysfunction‐associated diseases especially AD.

## 2 MATERIALS AND METHODS

### 2.1 Materials

#### 2.1.1 Collection and preparation of samples

African eggplant (*Solanum marcrocarpon* L) and Black nightshade (*Solanum nigrum* L) vegetables were purchased from local market in Ede, Osun State, Nigeria. Authentication of the samples was carried out at the Department of Biology, Federal University of Technology, Akure, Ondo State, Nigeria. The leaves were washed with water and chopped into small pieces, dried under shade, and milled into a fine powder using stainless steel electric blender. The pulverized samples were subsequently used for feed formulation.

#### 2.1.2 Chemicals and reagents

Chemicals and reagents used such as semicarbazide, benzylamine, acetylthiocholine iodide, and scopolamine were procured from Sigma‐Aldrich, Inc. (St Louis, MO); trichloroacetic acid (TCA) was sourced from Sigma‐Aldrich, Chemie GmbH (Steinheim, Germany); 2,4‐dinitrophenyl hydrazine (DNPH) from ACROS Organics (New Jersey, USA), methanol and acetic acid were sourced from BDH Chemicals Ltd. (Poole, England). All other chemicals were of analytical grade, while the water used for all analysis was glass distilled.

### 2.2 Methods

#### 2.2.1 Experimental animals

Albino rats weighing 150–200 g were used for this study. Rats were maintained at 25°C, on a 12‐hr light/12‐hr dark cycle. They were acclimatized under these conditions for 1–2 weeks before the experiment. The rats were allowed free access to food and water ad libitum. All applicable international, national, and/or institutional guidelines for the care and use of laboratory animals were followed.

#### 2.2.2 Experimental design

**Figure 1:**
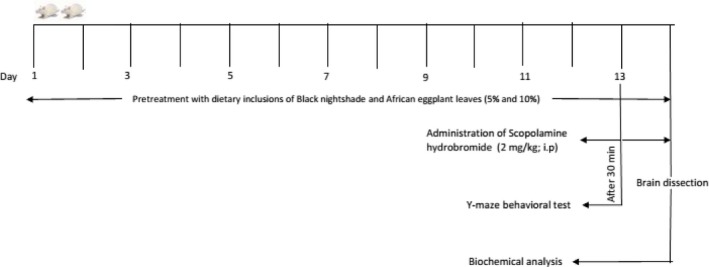
Experimental scheme for the effect of dietary inclusion of African eggplant and Black nightshade leaves on scopolamine‐induced amnesic rats

After a period of 2‐week acclimatization, the rats were randomly divided into seven groups (*n* = 4). Groups 1–III were fed basal diets, and groups IV and V were fed diets supplemented with 5% and 10% Black nightshade (BN) leaf inclusions, respectively, while groups VI and VII were fed diets supplemented with 5% and 10% African eggplant (AE) leaf inclusions, respectively, for 14 days. Animals in groups II–VII were administered scopolamine hydrobromide (2 mg/kg body weight; i.p) 30 min. to behavioral test (Sadek et al., 2016) and animals in group III serve as the positive control and were administered 5 mg/kg body weight (p.o.) of donepezil hydrochloride (Kamat et al., 2011) once before administration of scopolamine (see Figure 1 for the experimental protocol scheme).

#### 2.2.3 Feed formulation, sample inclusion, and treatment groups

Freshly formulated diets were prepared using the method reported by Ademiluyi, Oboh, Ogunsuyi, and Akinyemi (2012). All formulated feeds were kept in airtight containers at 4°C. Rats’ feeding per group includes the following:

Group 1: Normal control rats fed with basal diet (50% skimmed milk, 36% corn starch, 10% groundnut oil, and 4% vitamin and mineral premix).

Group II: Scopolamine‐administered (2 mg/kg body weight; i.p) rats fed with basal diet.

Group III: Positive control—Scopolamine‐administered rats fed with basal diet and administered donepezil (5 mg/kg body weight; p.o.).

Group IV: Scopolamine‐administered (2 mg/kg body weight) rats fed with diet supplemented with 5% Black nightshade leaves.

Group V: Scopolamine‐administered (2 mg/kg body weight) rats fed with diet supplemented with 10% Black nightshade leaves.

Group VI: Scopolamine‐administered (2 mg/kg body weight) rats fed with diet supplemented with 5% African eggplant leaves.

Group VII: Scopolamine‐administered (2 mg/kg body weight) rats fed with diet supplemented with 10% African eggplant leaves.

*Note*: skimmed milk = 16% protein. One gram of the mineral and vitamin premix = 3,200 iu vitamin A, 600 iu vitamin D3, 2.8 mg vitamin E, 0.6 mg vitamin K3, 0.8 mg vitamin B1, 1 mg vitamin B2, 6 mg niacin, 2.2 mg pantothenic acid, 0.8 mg vitamin B6, 0.004 mg vitamin B12, 0.2 mg folic acid, 0.1 mg biotin H2, 70 mg choline chloride, 0.08 mg cobalt, 1.2 mg copper, 0.4 mg iodine, 0.4 mg iron, 16 mg manganese, 0.08 mg selenium, 12.4 mg zinc, 0.5 mg antioxidant. Inclusions of AE and BN leaves were at equivalent weight basis.

### 2.3 Behavioral study

#### 2.3.1 Y‐maze (spontaneous alternation) test

This was carried out to analyze the spatial working memory of the animals. The Y‐maze test was performed according to a previously described method (Akinyemi et al., 2017). The maze was made of three identical arms, 40 cm long, 35 cm high, and 12 cm wide, positioned at equal angles and labeled A, B, and C. Rats were placed at the end of one arm and allowed to move freely through the maze during a 5‐min session. Spontaneous alternation was examined by visually recording the pattern of entrance into each arm in the maze for each rat. An arm entry was considered to be complete when the hind paws of the rat were completely placed on the arm. Alternation was defined as successive entries into the three arms on the overlapping triplet set (i.e., ABC, BCA…). Accordingly, the frequencies of alteration between the arms recorded were plotted to determine the memory index (percentage of correct alteration).

### 2.4 Bioassays

#### 2.4.1 Preparation of brain tissue homogenate

At the completion of Y‐maze test, the rats were immobilized by cervical dislocation and the whole‐brain tissues were rapidly isolated, rinsed with cold saline, placed on ice, and weighed. These tissues were subsequently homogenized in appropriate buffer (1:3 w/v) with about 10 up and down strokes at approximately 1,200 rev/min in a Teflon glass homogenizer (Mexxcare, mc14 362; Aayu‐shi Design Pvt. Ltd. India). The homogenates were centrifuged for 10 min at 3,000×*g* in a refrigerated centrifuge (KX3400C; KENXIN Intl. Co., Hong Kong) at 4°C to yield pellets that were discarded, and supernatants (homogenates), which were used for all bioassays.

#### 2.4.2 Determination of cholinesterases (ChEs) activity

The assay of ChEs (acetylcholinesterase [AChE] and butyrylcholinesterase [BChE]) activities was determined using the modified colorimetric method of Ellman, Courtney, Andres, and Featherstone (1961) as previously described by Akinyemi et al. (2016). The ChEs activities of the brain homogenates were determined in a 2‐ml final reaction mixture containing 0.1 mol/L phosphate buffer (pH 8.0), 3.3 mmol/L of 5,5′‐dithio‐bis(2‐nitrobenzoic) acid (DTNB), sample mixtures, and 0.1 mol/L phosphate buffer (pH 8.0). After incubation for 20 min at room temperature, the enzyme (40–50 μg of protein) pre‐incubated for 2 min. The reaction was subsequently initiated by adding the substrate (0.8 mmol/L acetylthiocholine iodide and butyrylthiocholine iodide for AChE and BChE, respectively). The absorbance of the mixture was monitored at 412 nm.

#### 2.4.3 Determination of monoamine oxidase activity

The activity of monoamine oxidase (MAO) was assayed according to a previously reported method (Green & Haughton, 1961) with slight modifications. In brief, the reaction mixture contained 0.025 mol/L phosphate buffer (pH 7.0), 0.0125 mol/L semicarbazide, 10 mmol/L benzylamine, and 100 μl of tissue homogenate. After 30‐min incubation, acetic acid was added and incubated for 3 min in boiling water bath followed by centrifugation. The resulting supernatant (1 ml) was mixed with equal volume of 2, 4‐Dinitrophenylhydrazine, and 1.25 ml of benzene was added after 10‐min incubation at room temperature. After separating the benzene layer, it was mixed with equal volume of 0.1 N NaOH. The alkaline layer was decanted and incubated at 80°C for 10 min. The orange–yellow color developed was measured at 450 nm in a UV/visible spectrophotometer (Jenway 6305 model).

#### 2.4.4 Determination of arginase activity

Arginase activity in the brain homogenate was determined by the method of Kaysen and Strecker (1973). In brief, the tissue homogenate (75 μl) was pre‐incubated for 5 min in a solution of 0.05 mmol/L Manganese chloride in 0.01 mmol/L Tris–HCl buffer (pH 7.5). Thereafter, the reaction was initiated by adding the reaction substrate (50 mmol/L L‐arginine) followed by incubation at 37°C for 10 min. The reaction was terminated by adding 2.5 ml of Ehrlich's reagent (p‐dimethylaminobenzaldehyde in 3.6 N HCl). The amount of urea produced was measured spectrophotometrically at 450 nm, normalized with protein, and used as an index for arginase activity.

#### 2.4.5 Estimation of nitrite level

Assay for brain nitrite level was carried out as an indicator of the amount of nitric oxide (NO) by a spectrophotometric assay using Griess reagent (0.1% *N*‐(1‐naphthyl) ethylenediamine dihydrochloride, 1% sulfanilamide, and 2.5% phosphoric acid) (Green et al., 1982). Equal volumes of tissue homogenate and Griess reagent were mixed, the mixture was incubated for 10 min at room temperature in the dark, and the absorbance was determined at 540 nm spectrophotometrically. The concentration of nitrite in the supernatant was determined from the sodium nitrite standard curve and expressed as μmol/mg protein.

#### 2.4.6 Lipid peroxidation assay

The lipid peroxidation assay was carried out according to the modified method of Ohakawa, Ohishi, and Yagi (1979). Briefly, 300 ml of the brain homogenate was added to 300 ml of 8.1% sodium dodecyl sulfate (SDS), 500 ml of acetic acid/HCl buffer (pH 3.4), and 500 ml of 0.8% thiobarbituric acid (TBA). This mixture was incubated at 100°C for 1 hr, and thiobarbituric acid reactive species (TBARS) produced were measured at 532 nm using a spectrophotometer. Malondialdehyde (MDA) was used as standard, and TBARS produced was reported as MDA equivalent.

#### 2.4.7 Antioxidant enzyme assays

Glutathione‐S‐transferase (GST) activity was assayed according to the method of Habig, Pabst, and Jakoby (1974), which involves the pre‐incubation of a reaction mixture containing 1.0 ml 100 mmol/L phosphate buffer (pH 6.5), 30 mmol/L 1‐chloro‐2,4‐dinitrobenzene (CDNB), and 0.7 ml of double distilled water for 5 min at 37°C. The reaction was started by the addition of 0.1 ml of the tissue homogenate and 0.1 ml 30 mmol/L glutathione as substrate. The absorbance of the reaction mixture was monitored after 5 min at 340 nm in a spectrophotometer and expressed as U/g protein. Superoxide dismutase (SOD) activity was determined by the method of Alia, Horcajo, Bravo, and Goya (2003), in which 0.05 ml of tissue homogenate was treated with 1.0 ml of 50 mmol/L carbonate buffer (pH 10.2) and 0.017 ml of adrenaline (0.06 mg/ml). The absorbance was read at 480 nm in spectrophotometer for 2 min at 15‐s intervals. SOD activity was expressed as U/g protein. Reduced glutathione (GSH) content was determined by the modified method of Ellman (1959). One milliliter of the supernatant was added to 0.5 ml of Ellman's reagent (19.8 mg of 5,5′ dithiobisnitrobenzoic acid in 100 ml of 0.1% sodium citrate) and 3.0 ml of 0.2 mol/L phosphate buffer (pH 8.0). The absorbance was read at 412 nm in a spectrophotometer. Total protein was measured according to Bradford (1976) using serum albumin as standard.

### 2.5 Data analysis

Results were expressed as the mean ± standard deviation (*SD*). Mean values were appropriately analyzed and compared using one‐way analysis of variance (ANOVA) followed by Newman–Keuls multiple range post hoc test; significance was accepted at *p* < .05; *p* < .01; *p* < .001. All statistical analysis was carried out using GraphPad Prism version 5.00 for Windows.

## 3 RESULT

**Figure 2:**
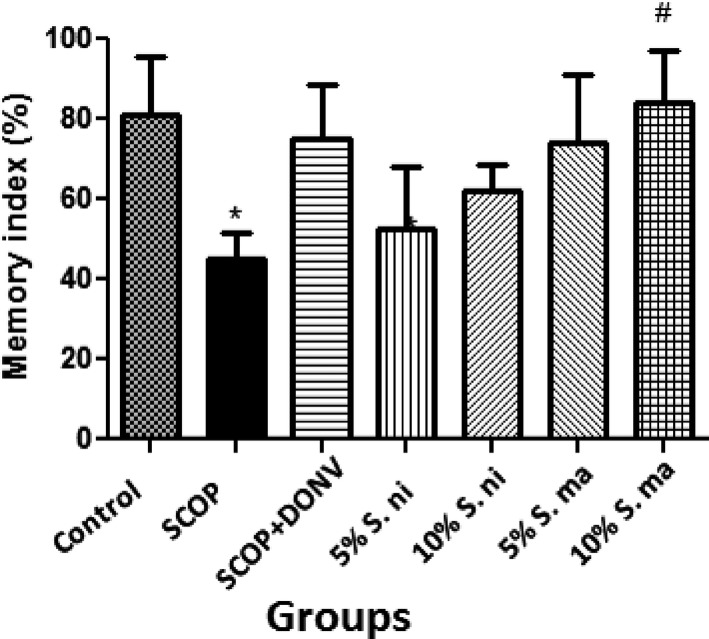
Spontaneous alternation (memory index) of scopolamine‐induced amnesic rats pretreated with dietary inclusions of African eggplant (*Solanum marcrocarpon* L) and Black nightshade (*Solanum nigrum* L) leaves. Values represent mean ± standard deviation (*n* = 4). Key: Control, Control rats fed with basal diet; SCOP, scopolamine‐administered (2 mg/kg body weight; i.p) rats fed with basal diet; SCOP+DON, Positive control—Scopolamine‐administered rats fed with basal diet and administered donepezil (5 mg/kg body weight; p.o.); 5% S. ni, scopolamine‐administered (2 mg/kg body weight) rats fed with diet supplemented with 5% Black nightshade (*Solanum nigrum* L) leaves; 10% S. ni = scopolamine‐administered (2 mg/kg body weight) rats fed with diet supplemented with 10% Black nightshade (*Solanum nigrum* L) leaves; 5% S. ma, scopolamine‐administered (2 mg/kg body weight) rats fed with diet supplemented with 5% African eggplant (*Solanum macrocarpon* L) leaves; 10% S. ma, scopolamine‐administered (2 mg/kg body weight) rats fed with diet supplemented with 10% African eggplant (*Solanum macrocarpon* L) leaves. *Mean values are significantly different (*p* < .05) compared to control rat group. ^#^Mean values are significantly different (*p* < .05) compared to scopolamine‐treated rat group

Investigations in this study commenced by assessing the effect of pretreatment with dietary inclusions of African eggplant (AE) and Black nightshade (BN) leaves of spatial working memory in scopolamine‐induced cognitive impaired rats monitored via the spontaneous alternation (Y‐maze) behavioral test. The result (Figure 2) revealed that 2 mg/kg scopolamine (SCOP) administered thirty minutes to this test significantly (*p* < .05) reduced the memory index in treated rats. However, pretreatment with dietary inclusions of AE and BN leaves ameliorated the impaired memory function, with dietary inclusion of 10% BN, is producing a significant (*p* < .05) restoration of the spatial working memory.

**Figure 3:**
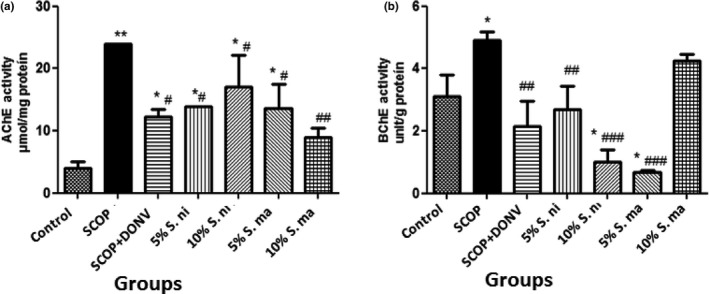
Effect of dietary inclusion of African eggplant (*Solanum marcrocarpon* L) and Black nightshade (*Solanum nigrum* L) leaves on (a) brain acetylcholinesterase (AChE) and (b) butyrylcholinesterase activities in scopolamine‐induced amnesic rats. Values represent mean ± *SD* (*n* = 4). Key: as described for Figure 2. *^,**^Mean values are significantly different (*p* < .05; *p* < .01) compared to control rat group. ^#,##,###^Mean values are significantly different (*p* < .05; *p* < .01; *p* < .001) compared to scopolamine‐treated rat group

**Figure 4:**
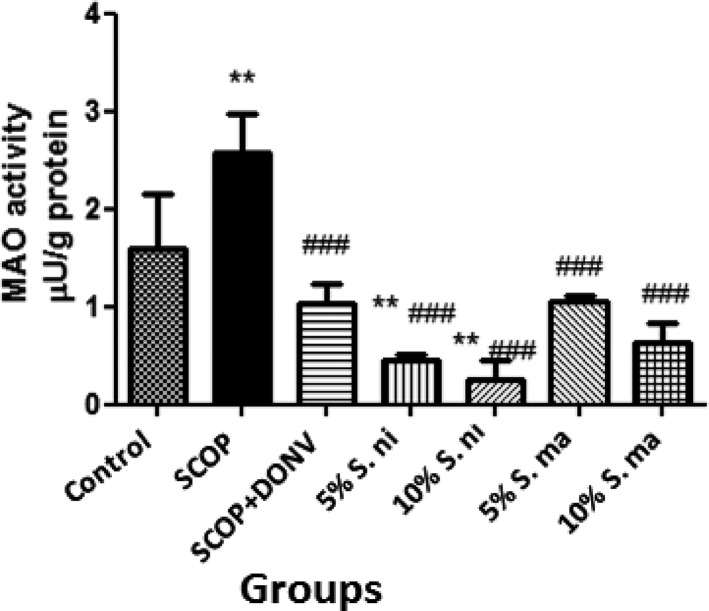
Effect of dietary inclusion of African eggplant (*Solanum marcrocarpon* L) and Black nightshade (*Solanum nigrum* L) leaves on brain monoamine oxidase (MAO) activity in scopolamine‐induced amnesic rats. Values represent mean ± *SD* (*n* = 4). Key: as described in Figure 2. **Mean values are significantly different (*p* < .01) compared to control rat group. ^###^Mean values are significantly different (*p* < .001) compared to scopolamine‐treated rat group

Figures 3a and b revealed the effect of dietary inclusions of AE and BN on brain AChE and butyrylcholinesterase (BChE) activities in scopolamine‐treated rats. According to the results, the activities of AChE and BChE were significantly increased (*p* < .05) in rats administered SCOP. However, pretreatment with dietary inclusions of AE and BN (5% and 10%) significantly (*p* < .05; *p* < .01) reversed the increase in AChE activity induced by SCOP, while all but 10% BN significantly attenuated the elevation in BChE activity induced by SCOP. Figure 4 depicts the effect of dietary inclusions of AE and BN (5% and 10%) on brain monoamine oxidase (MAO) activities in SCOP‐induced amnesic rats. This showed that there was significant elevation (*p* < .01) in MAO activity in SCOP‐induced rats, which was significantly reversed in rats pretreated with dietary inclusions of AE and BN (5% and 10%).

**Figure 5:**
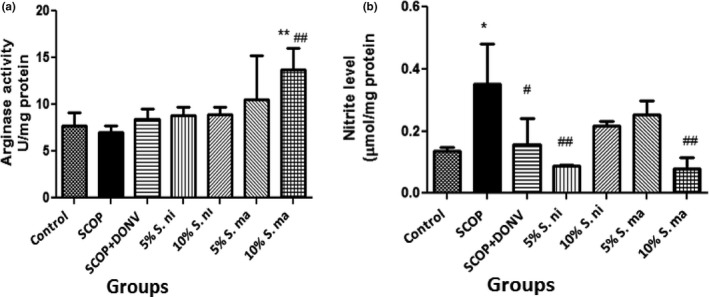
Effect of dietary inclusion of African eggplant (*Solanum marcrocarpon* L) and Black nightshade (*Solanum nigrum* L) leaves on (a) brain arginase activity and (b) nitrite level in scopolamine‐induced amnesic rats. Values represent mean ± *SD* (*n* = 4). Key: as described in Figure 2. *^,**^Mean values are significantly different (*p* < .05; *p* < .01) compared to control rat group. ^##^Mean values are significantly different (*p* < .01) compared to scopolamine‐treated rat group

The effect of dietary inclusions of AE and BN on brain arginase activity and nitrite content in SCOP‐treated rats was presented in Figure 5a and b, respectively. These showed that there was no significant difference (*p* > .05) in brain arginase activity among the various treatment groups, with the exception of SCOP‐treated rats pretreated with dietary inclusion of 10% AE which produced a significant (*p* < .01) elevated arginase activity. Furthermore, SCOP administration caused a significant (*p* < .05) increase in brain nitrite content in rats; these were, however, reversed in brain nitrite content in SCOP‐treated rats administered donepezil (DON), as well as those pretreated with dietary inclusions of AE and BN (5% and 10%).

**Figure 6:**
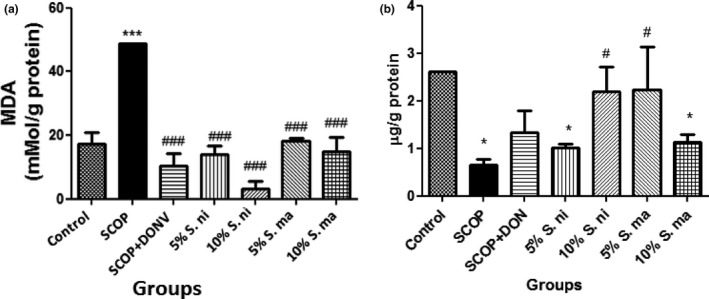
Effect of dietary inclusion of African eggplant (*Solanum marcrocarpon* L) and Black nightshade (*Solanum nigrum* L) leaves on brain (a) malondialdehyde (MDA) and (b) reduced glutathione (GSH) contents in scopolamine‐induced amnesic rats. Values represent mean ± *SD* (*n* = 4). Key: as described in Figure 2. *^,***^Mean values are significantly different (*p* < .05; *p* < .001) compared to control rat group. ^#,###^Mean values are significantly different (*p* < .05; *p* < .001) compared to scopolamine‐treated rat group

Furthermore, administration of SCOP induced a significant elevation (*p* < .001) in brain malondialdehyde (MDA) content (Figure 6a). However, pretreatment with dietary inclusions of AE and BN (5% and 10%) significantly (*p* < .001) reversed the elevation of MDA content induced by SCOP; this reversal was not significantly different (*p* > .05) from that observed in SCOP‐induced rats administered DON.

**Figure 7:**
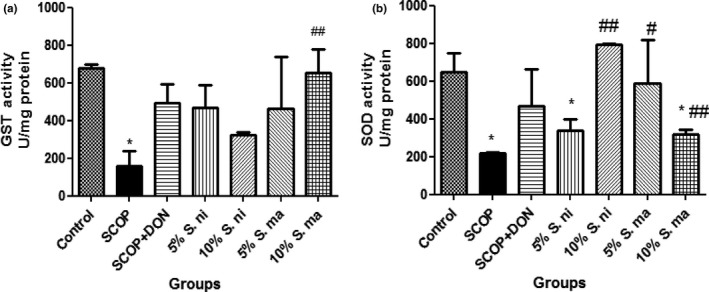
Effect of dietary inclusion of African eggplant (*Solanum marcrocarpon* L) and Black nightshade (*Solanum nigrum* L) leaves on brain (a) glutathione‐S‐transferase (GST) and (b) superoxide dismutase (SOD) activities in scopolamine‐induced amnesic rats. Values represent mean ± *SD* (*n* = 4). Key: as described in Figure 2. *Mean values are significantly different (*p* < .05) compared to control rat group. ^#,##^Mean values are significantly different (*p* < .05; *p* < .01) compared to scopolamine‐treated rat group

In Figure 6b, the effect of dietary inclusions of AE and BN (5% and 10%) on brain reduced glutathione (GSH) content was presented. This showed that pretreatment with dietary inclusions of AE and BN significantly (*p* < .05) reversed the depletion in brain GSH content induced by SCOP. In addition, as reported in Figure 7a and b, pretreatment with dietary inclusions of AE and BN (5% and 10%) ameliorated the reduction in the activities of brain glutathione‐S‐transferase (GST) and superoxide dismutase (SOD) induced by SCOP.

## 4 DISCUSSION

The ability of dietary inclusions of African eggplant (AE) and Black nightshade (BN) leaves (5% and 10%) to ameliorate the elevation of cholinesterases (ChEs) activities in scopolamine (SCOP)‐induced cognitive impaired rats could be one of the mechanisms behind its neuroprotective properties as reported in folklore. Neurodegenerative diseases such as AD are characterized by elevated ChEs activities which result in impaired cholinergic neurotransmission as a result of excessive hydrolysis of acetylcholine (Klafki et al., 2006; Nwanna et al., 2016). Previous findings have shown that SCOP can induce an elevation in rats’ AChE activity, thereby mimicking the pathophysiology of many neurodegenerative diseases (Gutierres et al., 2010; Xian et al., 2015). The ability of dietary inclusions of AE and BN leaves to reverse the SCOP‐induced elevation in ChEs activities in experimental rats could be as a result of their constituent phytochemicals. It has been previously reported that AE leaves are rich in phytochemicals such as alkaloids, sapogenins, and sterols (Gbile & Adesina, 1988). The plant is specifically known to be rich in glycoalkaloids especially solasodine which has been reported to promote neurogenesis (Patel, Singh, & Patel, 2013). Similarly, BN is specifically known to be rich in two alkaloids, solamargine and solasonine: the precursors of glycine solasodine which is of numerous pharmacologically importance (Liu et al., 2017). Interestingly, several findings have shown that plant‐derived alkaloids and alkaloid‐rich extracts have ChEs inhibitory properties (Ademiluyi, Ogunsuyi, Oboh, & Agbebi, 2016; Akomolafe, 2017; Akomolafe et al., 2017). Therefore, these constituent phytochemicals could be responsible for the observed reversal in the ChEs activities in SCOP‐induced amnesic rats.

Previous studies have correlated reduced cholinesterase activity with impaired learning and memory functions in different rat models (Adeniyi, Omatsuli, Akinyemi, & Ishola, 2016; Akinyemi et al., 2017; Rubio et al., 2017). In this study, we showed that scopolamine significantly reduced the spatial working memory of rats measured via the Y‐maze test. This agrees with previous reports that scopolamine induces cognitive deficiencies in animals (Akinyemi et al., 2017; Kwon et al., 2009; Soni & Parle, 2017). The Y‐maze test is an experimental behavioral procedure widely used to assess immediate spatial working memory, which is one of the indices of overall cognitive function (de Bruin et al., 2010; Hamlin, Windels, Boskovic, Sah, & Coulson, 2013). Therefore, the ability of pretreated AE and BN dietary inclusions to prevent SCOP‐induced impaired spatial working memory could be correlated with their anti‐ChEs activities.

Furthermore, it was observed in this study that dietary inclusions of AE and BN leaves (5% and 10%) significantly reversed the elevation of MAO activity in SCOP‐induced amnesic rats. It is noteworthy that previous reports show no significant effect of SCOP on brain MAO activity (Bihaqi, Singh, & Tiwari, 2011; Liu et al., 2017; Rubio et al., 2017); we, however, observed that these studies reported administration of 1 mg/kg SCOP. Nevertheless, El‐Khadragy et al. (2014) reported significant alterations in brain monoamines in rats administered 1.4 mg/kg SCOP. Thus, we hypothesize that the significant reduction in MAO activity induced by SCOP in this study could be as a result of the higher dose used (2 mg/kg). It has been reported that impairment of the monoaminergic system of neurotransmission resulting from elevated MAO activity contributes significantly to the pathogenesis and progression of several neurodegenerative diseases including AD and PD (Kaludercic, Carpi, Menabò, Di Lisa, & Paolocci, 2011; Nwanna et al., 2016). Elevation in MAO activity results in the rapid breakdown of both inhibitory and excitatory monoamine neurotransmitters such as adrenaline, dopamine, and serotonin, which are crucial in anxiety and mood disorders (Oboh et al., 2016; Thomas, 2000). Therefore, in the clinical management of several neurodegenerative diseases, especially AD and PD, MAO inhibitors are often useful therapies, especially as antidepressants (Baker, Matveychuk, MacKenzie, Dursun, & Mousseau, 2012; Oboh et al., 2016; Thomas, 2000; Yu, Kong, & Chen, 2002). Furthermore, several experimental studies have shown that plant extracts such as phenolic‐rich and alkaloid‐rich extracts elicited significant MAO inhibitory effects in both in vitro (Ademiluyi et al., 2016; Nwanna et al., 2016) and in vivo (Yu et al., 2002) studies. Therefore, the ability of dietary inclusions of AE and BN leaves to reverse elevation in MAO activities in SCOP‐induced amnesic rats could also be attributed to the constituent phytochemicals in the vegetable with bioactive properties.

We quantified the brain nitrite level in this study as a measure of the nitric oxide (NO) content. In agreement with earlier studies (El‐Khadragy et al., 2014; Giridharan, Thandavarayan, Sato, Ko, & Konishi, 2011), we observed that SCOP caused a significant elevation in brain NO content. NO can serve as a neurotransmitter that mediates synaptic activity, neural plasticity, and memory functions (Pitsikas, 2015). Furthermore, NO has been shown to influence presynaptic cellular transmission processes and promotes synaptic plasticity (Pitsikas, 2015). Behavioral studies have reported that inhibition of nitric oxide synthase (NOS), which synthesizes NO, impairs learning and such deficiencies could be attenuated by NO donors (Paul & Ekambaram, 2011). Therefore, the ability of dietary inclusions of AE and BN to reverse the elevation in NO content induced by SCOP is also noteworthy and could be another neuroprotective mechanism. This is more so because the neuronal actions of NO are concentration dependent, with low concentrations eliciting neuroprotective properties and mediating neurotransmission, while higher concentrations are involved in immune responses and inflammatory actions, and are considered neurotoxic (Calabrese et al., 2007; Pitsikas, 2015). In addition, the ability of BN and AE to prevent NO depletion could also justify the improved cognitive function in treated rats. This is so because a connection has been established between depletion of a neuronal NO through inhibition of its synthesis, reduction in acetylcholine release, and impairment in conditional responses in rats (Paul & Ekambaram, 2011).

Oxidative stress is one of the major risk factors for the pathogenesis and progression of several neurodegenerative diseases, through mechanisms such as free radical‐induced mitochondrial dysfunction and neuronal cell death (Bhat et al., 2015; Lin & Beal, 2006; Shukla, Mishra, & Pant, 2011). Furthermore, elevation in MAO activities has been linked to free radical‐induced oxidative stress. This is because hydrogen peroxide (H_2_O_2_) is one of the by‐products of MAO hydrolysis of biogenic amines (Cadenas & Davies, 2000). In the presence of Fe^2+^, H_2_O_2_ participates in the Fenton reaction to form the highly reactive hydroxyl (OH) radical which can cause oxidation of biomolecules including lipids, proteins, and DNA (Yamaguchi, Ariga, Yoshimura, & Nakazawa, 2000). This study revealed that there was a significant increase in brain malondialdehyde (MDA) content in SCOP‐treated rats. MDA is one of the products of the lipid peroxidation chain reaction, and it has been extensively used to quantify the extent of lipid peroxidation in any biological tissue. However, the dietary inclusions of AE and BN leaves (5% and 10%) significantly prevented the elevation in brain MDA contents. One of the justifications for this observation could be as a result of the ability of dietary inclusions of AE and BN leaves to significantly reduce MAO activities in SCOP‐induced amnesic rats as earlier discussed. Secondly, the antioxidant properties of AE and BN leaves could have also contributed to their abilities to reverse elevation in MDA content in SCOP‐treated rats. Previous studies (Ademiluyi, Oboh, Aragbaiye, Oyeleye, & Ogunsuyi, 2015; Oboh, Akinyemi, Ademiluyi, & Bello, 2014) have shown that green leafy vegetables rich in antioxidant phytochemicals are capable of inhibiting lipid peroxidation chain reactions.

Impairments in endogenous antioxidant defense systems such as catalase, glutathione (GSH), superoxide dismutase (SOD), and glutathione‐S‐transferase (GST) are possible consequences of oxidative stress. To further corroborate the antioxidant properties of dietary inclusions of AE and BN leaves, this study showed that there was a significant elevation in brain GSH contents, which was observed to be reduced significantly in SCOP‐treated rats. Similarly, SCOP‐induced rats pretreated with dietary inclusions of AE and AN also exhibited elevated activities of GST and SOD which were reduced significantly in SCOP‐induced rats. GSH is a ubiquitous endogenous antioxidant molecule that functions to stabilize free radicals by serving as proton donors, particularly against hydroxyl (OH) radicals (Bains & Shaw, 1997). Previous studies have shown an age‐related decline in GSH to be a key factor in the aging process which could contribute to several changes observed in the normal aging process as well as in the pathogenesis of various diseases (Bains & Shaw, 1997). On the other hand, GST functions to conjugate GSH to xenobiotics for the purpose of detoxification (Sofia, Segura‐Aguilar, Widersten, Johansson, & Mannervik, 1997). Therefore, the increase in GST activity in SCOP‐induced rats pretreated with dietary inclusions of AE and BN (5% and 10%) could be another mechanism by which AE and BN help overcome SCOP‐induced neurotoxicity by increasing the activity of GST detoxifying enzyme. The ability of dietary inclusions of AE and AN (5% and 10%) to significantly reverse the reduction in both GSH contents and GST and SOD activities in SCOP‐induced rats could therefore contribute significantly to the neuroprotective properties of these vegetables.

## 5 CONCLUSION

The ability of dietary inclusions of African eggplant (*Solanum marcrocarpon* L) and Black nightshade (*S. nigrum* L) leaves in ameliorating scopolamine‐induced cognitive impairment in rats was reported in this study. Scopolamine‐administered rats pretreated with dietary inclusions of African eggplant and Black nightshade leaves (5% and 10%) were able to significantly reverse the impairments in critical enzymes of the cholinergic (AChE and BChE) and monoaminergic (MAO) systems, as well as the antioxidant system induced by scopolamine. These therapeutic properties could be attributed to the constituent phytochemicals in the vegetable. Therefore, this study has provided some scientific rationale for these vegetables in the management of neurodegenerative diseases as obtained in folklore. Hence Therefore, these vegetables could serve as a potential source of functional foods and nutraceuticals for the prevention and management of neurodegenerative diseases, especially AD.

## CONFLICT OF INTEREST

The authors have no conflict of interest to declare.

## References

[fsn3628-bib-0001] Ademiluyi, A. O. , Oboh, G. , Aragbaiye, F. P. , Oyeleye, S. I. , & Ogunsuyi, O. B. (2015). Antioxidant properties and in vitro α‐amylase and α‐glucosidase inhibitory properties of phenolics constituents from different varieties of *Corchorus* spp. Journal of Taibah University Medical Sciences, 10(3), 278–287. https://doi.org/10.1016/j.jtumed.2014.11.005

[fsn3628-bib-0002] Ademiluyi, A. O. , Oboh, G. , Ogunsuyi, O. B. , & Akinyemi, A. J. (2012). Attenuation of gentamycin‐induced nephrotoxicity in rats by dietary inclusion of ginger (*Zingiber officinale*) and turmeric (*Curcuma longa*) rhizomes. Nutrition and Health, 21(4), 209–218. https://doi.org/10.1177/0260106013506668 24197862 10.1177/0260106013506668

[fsn3628-bib-0003] Ademiluyi, A. O. , Ogunsuyi, O. B. , Oboh, G. , & Agbebi, O. J. (2016). Jimson weed (*Datura stramonium*). Comparative Clinical Pathology, 25(4), 733–741. https://doi.org/10.1007/s00580-016-2257-6

[fsn3628-bib-0004] Adeniyi, P. A. , Omatsuli, E. P. , Akinyemi, A. J. , & Ishola, A. O. (2016). Caffeine plus nicotine improves motor function, spatial and non‐spatial working memory and functional indices in BALB/c male mice. Pathophysiology, 23(4), 251–258. https://doi.org/10.1016/j.pathophys.2016.08.002 27596362 10.1016/j.pathophys.2016.08.002

[fsn3628-bib-0005] Akinyemi, A. J. , Oboh, G. , Oyeleye, S. I. , & Ogunsuyi, O. (2017). Anti‐amnestic effect of curcumin in combination with donepezil, an anticholinesterase drug: Involvement of cholinergic system. Neurotoxicity Research, 31(4), 560–569. https://doi.org/10.1007/s12640-017-9701-5 28102474 10.1007/s12640-017-9701-5

[fsn3628-bib-0006] Akinyemi, A. J. , Throme, G. R. , Morsch, V. M. , Stefanello, N. , da Costa, P. , Cardos, A. , … Schetinger, M. R. C. (2016). Effect of dietary supplementation of ginger and turmeric rhizomes on ectonucleosidase, adenosine deminase and acetylcholineesterase in synaptosomes from the cerebral cortex of hypertensive rats. Journal of Applied Biomedicine, 14, 59–70. https://doi.org/10.1016/j.jab.2015.06.001

[fsn3628-bib-0007] Akomolafe, S. F. (2017). The effects of caffeine, caffeic acid, and their combination on acetylcholinesterase, adenosine deaminase and arginase activities linked with brain function. Journal of Food Biochemistry, 41, e12401 https://doi.org/10.1111/jfbc.12401

[fsn3628-bib-0008] Akomolafe, S. F. , Akinyemi, A. J. , Ogunsuyi, O. B. , Oyeleye, S. I. , Oboh, G. , Adeoyo, O. O. , & Allismith, Y. R. (2017). Effect of caffeine, caffeic acid and their various combinations on enzymes of cholinergic, monoaminergic and purinergic systems critical to neurodegeneration in rat brain—In vitro. NeuroToxicology, 62, 6–13. https://doi.org/10.1016/j.neuro.2017.04.008 28465162 10.1016/j.neuro.2017.04.008

[fsn3628-bib-0009] Alia, M. , Horcajo, C. , Bravo, L. , & Goya, L. (2003). Effect of grape antioxidant dietary fiber on the total antioxidant capacity and the activity of liver antioxidant enzymes in rat. Nutrition Research, 23, 1251–1267. https://doi.org/10.1016/S0271-5317(03)00131-3

[fsn3628-bib-0010] Bains, J. S. , & Shaw, C. A. (1997). Neurodegenerative disorders in humans: The role of glutathione in oxidative stress‐mediated neuronal death. Brain Research Reviews, 25(3), 335–358. https://doi.org/10.1016/S0165-0173(97)00045-3 9495562 10.1016/s0165-0173(97)00045-3

[fsn3628-bib-0011] Baker, G. B. , Matveychuk, D. , MacKenzie, E. M. , Dursun, S. M. , & Mousseau, D. D. (2012). Monoamine oxidase inhibitors and neuroprotective mechanisms. Bulletin of Clinical Psychopharmacology, 22, 293–296. https://doi.org/10.5455/bcp.20121030014051

[fsn3628-bib-0012] Baradaran, A. , Rabiei, Z. , Rafieian, M. , & Shirzad, H. (2012). A review study on medicinal plants affecting amnesia through cholinergic system. Journal of HerbMed Pharmacology, 1(1), 3–9.

[fsn3628-bib-0013] Bhat, A. H. , Dar, K. B. , Anees, S. , Zargar, M. A. , Masood, A. , Sofi, M. A. , & Ganie, S. A. (2015). Oxidative stress, mitochondrial dysfunction and neurodegenerative diseases; a mechanistic insight. Biomedicine & Pharmacotherapy, 74, 101–110. https://doi.org/10.1016/j.biopha.2015.07.025 26349970 10.1016/j.biopha.2015.07.025

[fsn3628-bib-0014] Bihaqi, S. W. , Singh, A. P. , & Tiwari, M. (2011). In vivo investigation of the neuroprotective property of *Convolvulus pluricaulis* in scopolamine‐induced cognitive impairments in Wistar rats. Indian Pharmacological Society, 43(5), 520 https://doi.org/10.4103/0253-7613.84958 10.4103/0253-7613.84958 PMC3195120 22021993

[fsn3628-bib-0015] Bradford, M. M. (1976). A rapid and sensitive method for the quantitation of microgram quantities of protein utilizing the principle of protein‐dye binding. Analytical Biochemistry, 72(1–2), 248–254. https://doi.org/10.1016/0003-2697(76)90527-3 942051 10.1016/0003-2697(76)90527-3

[fsn3628-bib-0016] de Bruin, N. M. W. J. , Prickaerts, J. , Lange, J. H. , Akkerman, S. , Andriambeloson, E. , de Haan, M. , … Kruse, C. G. (2010). SLV330, a cannabinoid CB1 receptor antagonist, ameliorates deficits in the T‐maze, object recognition and social recognition tasks in rodents. Neurobiology of Learning and Memory, 93, 522–531. https://doi.org/10.1016/j.nlm.2010.01.010 20132903 10.1016/j.nlm.2010.01.010

[fsn3628-bib-0017] Cadenas, E. , & Davies, K. J. (2000). Mitochondrial free radical generation, oxidative stress, and aging. Free Radical Biology and Medicine, 29(3), 222–230. https://doi.org/10.1016/S0891-5849(00)00317-8 11035250 10.1016/s0891-5849(00)00317-8

[fsn3628-bib-0018] Calabrese, V. , Guagliano, E. , Sapienza, M. , Panebianco, M. , Calafato, S. , Puleo, E. , … Stella, A. G. (2007). Redox regulation of cellular stress response in aging and neurodegenerative disorders: Role of vitagenes. Neurochemical Research, 32(4–5), 757–773. https://doi.org/10.1007/s11064-006-9203-y 17191135 10.1007/s11064-006-9203-y

[fsn3628-bib-0019] Duthey, B. (2013). Priority medicines for Europe and the world: A public health approach to innovation. WHO Background paper 6.

[fsn3628-bib-0020] El‐Khadragy, M. , Al‐Olayan, E. E. , & Abdel Moneim, E. A. (2014). Neuroprotective effects of citrus reticulata in scopolamine‐induced dementia oxidative stress in rats. CNS & Neurological Disorders – Drug Targets, 13(4), 684–690. https://doi.org/10.2174/1871527313666140618105404 24938777 10.2174/1871527313666140618105404

[fsn3628-bib-0021] Ellman, G. I. (1959). Tissue sulfhydryl groups. Archives of Biochemistry and Biophysics, 82, 70–74. https://doi.org/10.1016/0003-9861(59)90090-6 13650640 10.1016/0003-9861(59)90090-6

[fsn3628-bib-0022] Ellman, G. L. , Courtney, K. D. , Andres Jr, V. , & Featherstone, R. M. (1961). A new and rapid cholimetric determination of acetylcholinesterase activity. Biochemical Pharmacology, 7, 88–95. https://doi.org/10.1016/0006-2952(61)90145-9 13726518 10.1016/0006-2952(61)90145-9

[fsn3628-bib-0023] Gbile, Z. O. , & Adesina, S. K. (1988). Nigerian Solanum species of economic importance. Annals of the Missouri Botanical Garden, 75, 862–865. https://doi.org/10.2307/2399374

[fsn3628-bib-0024] Giridharan, V. V. , Thandavarayan, R. A. , Sato, S. , Ko, K. M. , & Konishi, T. (2011). Prevention of scopolamine‐induced memory deficits by schisandrin B, an antioxidant lignan from *Schisandra chinensis* in mice. Free Radical Research, 45(8), 950–958. https://doi.org/10.3109/10715762.2011.571682 21615274 10.3109/10715762.2011.571682

[fsn3628-bib-0025] Goverdhan, P. , Sravanthi, A. , & Mamatha, T. (2012). Neuroprotective effects of meloxicam and selegiline in scopolamine‐induced cognitive impairment and oxidative stress. International Journal of Alzheimer's Disease, 2012, 1–8. https://doi.org/10.1155/2012/974013 10.1155/2012/974013 PMC3320018 22536538

[fsn3628-bib-0026] Green, A. L. , & Haughton, T. M. (1961). Colorimetric method for the estimation of monoamine oxidase. Biochemical Journal, 78, 172 https://doi.org/10.1042/bj0780172 13708157 10.1042/bj0780172 PMC1205191

[fsn3628-bib-0027] Green, L. C. , Wagner, D. A. , Glogowski, J. , Skipper, P. L. , Wishnok, J. S. , & Tannenbaum, S. R. (1982). Analysis of nitrate, nitrite, and [15N] nitrate in biological fluids. Analytical Biochemistry, 126, 131–138. https://doi.org/10.1016/0003-2697(82)90118-X 7181105 10.1016/0003-2697(82)90118-x

[fsn3628-bib-0028] Gutierres, J. M. , Carvalho, F. B. , Schetinger, M. R. C. , Agostinho, P. , Marisco, P. C. , Vieira, J. M. , … Mazzanti, C. M. (2010). Neuroprotective effect of anthocyanins on acetylcholinesterase activity and attenuation of scopolamine‐induced amnesia in rats. The International Journal of Developmental Neuroscience, 33, 88–97. 10.1016/j.ijdevneu.2013.12.006 24374256

[fsn3628-bib-0029] Habig, W. H. , Pabst, M. J. , & Jakoby, W. B. (1974). Glutathione S‐transferases: The first enzymatic step in mercapturic acid and formation. Journal of Biological Chemistry, 249, 7130–7139. 4436300

[fsn3628-bib-0030] Haliński, L. P. , Paszkiewicz, M. , Gołębiowski, M. , & Stepnowski, P. (2012). The chemical composition of cuticular waxes from leaves of the gboma eggplant (*Solanum macrocarpon* L.). Journal of Food Compositions and Analysis, 25, 74–78. https://doi.org/10.1016/j.jfca.2011.06.004

[fsn3628-bib-0031] Hamlin, A. S. , Windels, F. , Boskovic, Z. , Sah, P. , & Coulson, E. J. (2013). Lesions of the basal forebrain cholinergic system in mice disrupt idiothetic navigation. PLoS ONE, 8, 53472 https://doi.org/10.1371/journal.pone.0053472 10.1371/journal.pone.0053472 PMC3540070 23320088

[fsn3628-bib-0032] Imbimbo, B. P. , Lombard, J. , & Pomara, N. (2005). Pathophysiology of Alzheimer's disease. Neuroimaging Clinics of North America, 15(4), 727–753. https://doi.org/10.1016/j.nic.2005.09.009 16443487 10.1016/j.nic.2005.09.009

[fsn3628-bib-0033] Jain, R. , Sharma, A. , Gupta, S. , Sarethy, I. P. , & Gabrani, R. (2011). *Solanum nigrum*: Current perspectives on therapeutic properties. Alternative Medicine Review, 16, 78–85. 21438649

[fsn3628-bib-0034] Kaludercic, N. , Carpi, A. , Menabò, R. , Di Lisa, L. , & Paolocci, N. (2011). Monoamine oxidases (MAO) in the pathogenesis of heart failure and ischemia/reperfusion injury. Biochimica et Biophysica Acta, 1813(7), 1323–1332. https://doi.org/10.1016/j.bbamcr.2010.09.010 20869994 10.1016/j.bbamcr.2010.09.010 PMC3030628

[fsn3628-bib-0035] Kamat, P. K. , Tota, S. , Shukla, R. , Ali, S. , Najmi, A. K. , & Nath, C. (2011). Mitochondrial dysfunction: A crucial event in okadaic acid (ICV) induced memory impairment and apoptotic cell death in rat brain. Pharmacology, Biochemistry and Behavior, 100(2), 311–319. https://doi.org/10.1016/j.pbb.2011.08.019 10.1016/j.pbb.2011.08.019 21893081

[fsn3628-bib-0036] Kaysen, G. A. , & Strecker, H. J. (1973). Increased arginase activity levels caused by nitric oxide synthase dysfunction. The New England Journal of Medicine, 323, 1234–1238.

[fsn3628-bib-0037] Klafki, H. W. , Staufenbiel, M. , Kornhuber, J. , & Wiltfang, J. (2006). Therapeutic approaches to Alzheimer's disease. Brain, 129(11), 2840–2855. https://doi.org/10.1093/brain/awl280 17018549 10.1093/brain/awl280

[fsn3628-bib-0038] Kwon, S. H. , Kim, H. C. , Lee, S. Y. , & Jang, C. G. (2009). Loganin improves learning and memory impairments induced by scopolamine in mice. European Journal of Pharmacology, 619, 44–49. https://doi.org/10.1016/j.ejphar.2009.06.062 19666019 10.1016/j.ejphar.2009.06.062

[fsn3628-bib-0039] Lin, M. T. , & Beal, M. F. (2006). Mitochondrial dysfunction and oxidative stress in neurodegenerative diseases. Nature, 443(7113), 787 https://doi.org/10.1038/nature05292 17051205 10.1038/nature05292

[fsn3628-bib-0040] Liu, W. , Rabinovich, A. , Nash, Y. , Frenkel, D. , Wang, Y. , Youdim, M. B. , & Weinreb, O. (2017). Anti‐inflammatory and protective effects of MT‐031, a novel multitarget MAO‐A and AChE/BuChE inhibitor in scopolamine mouse model and inflammatory cells. Neuropharmacology, 113, 445–456. https://doi.org/10.1016/j.neuropharm.2016.10.028 27984078 10.1016/j.neuropharm.2016.10.028

[fsn3628-bib-0041] Marisco, P. C. , Carvalho, F. B. , Rosa, M. M. , Girardi, B. A. , Gutierres, J. M. , Jaques, J. A. A. , … Rubin, M. A. (2013). Piracetam prevents scopolamine‐induced memory impairment and decrease of NTPDase, 5′‐nucleotidase and adenosine deaminase activities. Neurochemical Research, 38, 1704–1714. https://doi.org/10.1007/s11064-013-1072-6 23677777 10.1007/s11064-013-1072-6

[fsn3628-bib-0042] Martin, J. B. (1999). Molecular basis of the neurodegenerative disorders. The New England Journal of Medicine, 340, 1970–1980. https://doi.org/10.1056/NEJM199906243402507 10379022 10.1056/NEJM199906243402507

[fsn3628-bib-0043] Nieoullon, A. (2011). Neurodegenerative diseases and neuroprotection: Current views and prospects. Journal of Applied Biomedicine, 9, 173–183. https://doi.org/10.2478/v10136-011-0013-4

[fsn3628-bib-0044] Nwanna, E. E. , Oyeleye, S. I. , Ogunsuyi, O. B. , Oboh, G. , Boligon, A. A. , & Athayde, M. L. (2016). In vitro neuroprotective properties of some commonly consumed green leafy vegetables in Southern Nigeria. NFS Journal, 2, 19–24. https://doi.org/10.1016/j.nfs.2015.12.002

[fsn3628-bib-0045] Oboh, G. , Akinyemi, A. J. , Ademiluyi, A. O. , & Bello, F. O. (2014). Inhibitory effect of some tropical green leafy vegetables on key enzymes linked to Alzheimer's disease and some pro‐oxidant induced lipid peroxidation in rats’ brain. Journal of Food Science and Technology, 51(5), 884–891. https://doi.org/10.1007/s13197-011-0572-0 24803694 10.1007/s13197-011-0572-0 PMC4008742

[fsn3628-bib-0046] Oboh, G. , Ekperigin, M. M. , & Kazeem, M. I. (2005). Nutritional and haemolytic properties of eggplants (*Solanum macrocarpon*) leaves. Journal of Food Compositions and Analysis, 18, 153–160. https://doi.org/10.1016/j.jfca.2003.12.013

[fsn3628-bib-0047] Oboh, G. , Nwanna, E. E. , Oyeleye, S. I. , Olasehinde, T. A. , Ogunsuyi, O. B. , & Boligon, A. A. (2016). In vitro neuroprotective potentials of aqueous and methanol extracts from *Heinsia crinita* leaves. Food Science and Human Wellness, 5(2), 95–102. https://doi.org/10.1016/j.fshw.2016.03.001

[fsn3628-bib-0048] Oboh, G. , Ogunsuyi, O. B. , & Olonisola, O. E. (2017). Does caffeine influence the anticholinesterase and antioxidant properties of donepezil? Evidence from in vitro and in vivo studies. Metabolic Brain Disease, 32(2), 629–639. https://doi.org/10.1007/s11011-017-9951-1 28091956 10.1007/s11011-017-9951-1

[fsn3628-bib-0049] Ogunsuyi, O. B. , Adeoyo, O. O. , & Oboh, G. (2017). Food‐drug Interaction: New paradigm in therapeutic potentials of functional food In Oboh G. (Ed.), Functional foods: Unlocking the medicine in foods (pp. 153–159). Akure, Nigeria: Graceland Prints. ISBN: 978‐978‐38530‐0‐7.

[fsn3628-bib-0050] Ohakawa, H. , Ohishi, N. , & Yagi, K. (1979). Assay of lipid peroxidation in animal tissue by thiobarbituric acid reaction. Analytical Biochemistry, 95, 351–358. https://doi.org/10.1016/0003-2697(79)90738-3 36810 10.1016/0003-2697(79)90738-3

[fsn3628-bib-0051] Patel, K. , Singh, R. B. , & Patel, D. K. (2013). Medicinal significance, pharmacological activities, and analytical aspects of solasodine: A concise report of current scientific literature. Journal of Acute Disease, 2(2), 92–98. https://doi.org/10.1016/S2221-6189(13)60106-7

[fsn3628-bib-0052] Paul, V. , & Ekambaram, P. (2011). Involvement of nitric oxide in learning & memory processes. Indian Journal of Medical Research, 133(5), 471. 21623030 PMC3121276

[fsn3628-bib-0053] Pitsikas, N. (2015). The role of nitric oxide in the object recognition memory. Behavioural Brain Research, 285, 200–207. https://doi.org/10.1016/j.bbr.2014.06.008 24933185 10.1016/j.bbr.2014.06.008

[fsn3628-bib-0054] Reitz, C. , Brayne, C. , & Mayeux, R. (2011). Epidemiology of Alzheimer disease. Nature Reviews Neurology, 7(3), 137–152. https://doi.org/10.1038/nrneurol.2011.2 21304480 10.1038/nrneurol.2011.2 PMC3339565

[fsn3628-bib-0055] Rubio, J. , Dang, H. , Gong, M. , Liu, X. , Chen, S. L. , & Gonzales, G. F. (2017). Aqueous and hydroalcoholic extracts of Black Maca (*Lepidium meyenii*) improve scopolamine‐induced memory impairment in mice. Food and Chemical Toxicology, 45(10), 1882–1890. 10.1016/j.fct.2007.04.002 17543435

[fsn3628-bib-0056] Sadek, B. , Khan, N. , Darras, F. H. , Pockes, S. , & Decker, M. (2016). The dual‐acting AChE inhibitor and H 3 receptor antagonist UW‐MD‐72 reverses amnesia induced by scopolamine or dizocilpine in passive avoidance paradigm in rats. Physiology & Behavior, 165, 383–391. https://doi.org/10.1016/j.physbeh.2016.08.022 27568232 10.1016/j.physbeh.2016.08.022

[fsn3628-bib-0057] Sánchez‐Mata, M. C. , Yokoyama, W. E. , Hong, Y. J. , & Prohens, J. (2010). α‐Solasonine and α‐solamargine contents of gboma (*Solanum macrocarpon* L.) and scarlet (*Solanum aethiopicum* L.) eggplants. Journal of Agriculture and Food Chemistry, 58, 5502–5508. https://doi.org/10.1021/jf100709g 10.1021/jf100709g 20397650

[fsn3628-bib-0058] Shukla, V. , Mishra, S. K. , & Pant, H. C. (2011). Oxidative stress in neurodegeneration. Advances in Pharmacological Sciences, 2011, 1–13. https://doi.org/10.1155/2011/572634 10.1155/2011/572634 PMC3177364 21941533

[fsn3628-bib-0059] Sofia, B. A. E. Z. , Segura‐Aguilar, J. , Widersten, M. , Johansson, M. S. , & Mannervik, B. (1997). Glutathione transferases catalyse the detoxication of oxidized metabolites (o‐quinones) of catecholamines and may serve as an antioxidant system preventing degenerative cellular processes. Biochemical Journal, 324(1), 25–28. 9164836 10.1042/bj3240025 PMC1218396

[fsn3628-bib-0065] Soni, K. , & Parle, M. (2017). Trachyspermum ammi seeds supplementation helps reverse scopolamine, alprazolam and electroshock induced amnesia. Neurochemical Research, 42(5), 1333–1344. 28097466 10.1007/s11064-017-2177-0

[fsn3628-bib-0060] Thomas, T. (2000). Monoamine oxidase‐B inhibitors in the treatment of Alzheimers disease. Neurobiology of Aging, 21(2), 343–348. https://doi.org/10.1016/S0197-4580(00)00100-7 10867219 10.1016/s0197-4580(00)00100-7

[fsn3628-bib-0061] Xian, Y. F. , Ip, S. P. , Mao, Q. Q. , Su, Z. R. , Chen, J. N. , Lai, X. P. , & Lin, Z.‐X. (2015). Honokiol improves learning and memory impairments induced by scopolamine in mice. The European Journal of Pharmacology, 760, 88–95. https://doi.org/10.1016/j.ejphar.2015.04.013 25912802 10.1016/j.ejphar.2015.04.013

[fsn3628-bib-0062] Yamaguchi, F. , Ariga, T. , Yoshimura, Y. , & Nakazawa, H. (2000). Antioxidative and anti‐glycation activity of garcinol from *Garcinia indica* fruit rind. Journal of Agricultural and Food Chemistry, 48(2), 180–185. https://doi.org/10.1021/jf990845y 10691613 10.1021/jf990845y

[fsn3628-bib-0063] Yu, Z. F. , Kong, L. D. , & Chen, Y. (2002). Antidepressant activity of aqueous extracts of *Curcuma longa* in mice. Journal of Ethnopharmacology, 83(1), 161–165. https://doi.org/10.1016/S0378-8741(02)00211-8 12413724 10.1016/s0378-8741(02)00211-8

